# Food density drives diet shift of the invasive mysid shrimp, *Limnomysis benedeni*

**DOI:** 10.1002/ece3.11202

**Published:** 2024-04-01

**Authors:** Varsha Rani, Zsófia Horváth, Jens C. Nejstgaard, Ádám Fierpasz, Károly Pálffy, Csaba F. Vad

**Affiliations:** ^1^ Institute of Aquatic Ecology HUN‐REN Centre for Ecological Research Budapest Hungary; ^2^ Doctoral School of Biology, Institute of Biology ELTE, Eötvös Loránd University Budapest Hungary; ^3^ Department of Plankton and Microbial Ecology Leibniz Institute of Freshwater Ecology and Inland Fisheries Stechlin Germany

**Keywords:** density‐dependent foraging, diet shift, food selectivity, invasive species, *Limnomysis benedeni*

## Abstract

Understanding the diet preferences and food selection of invasive species is crucial to better predict their impact on community structure and ecosystem functioning. *Limnomysis benedeni*, a Ponto‐Caspian invasive mysid shrimp, is one of the most successful invaders in numerous European river and lake ecosystems. While existing studies suggest potentially strong trophic impact due to high predation pressure on native plankton communities, little is known of its food selectivity between phyto‐ and zooplankton, under different food concentrations. Here, we therefore investigated the feeding selectivity of *L*. *benedeni* on two commonly occurring prey organisms in freshwaters, the small rotifer zooplankton *Brachionus calyciflorus* together with the microphytoplankton *Cryptomonas* sp. present in increasing densities. Our results demonstrated a clear shift in food selection, with *L. benedeni* switching from *B. calyciflorus* to *Cryptomonas* sp. already when the two prey species were provided in equal biomasses. Different functional responses were observed for the two food types, indicating somewhat different foraging mechanisms for each food type. These findings provide experimental evidence on the feeding flexibility of invasive mysid shrimps and potential implications for trophic interactions in invaded ecosystems.

## Graphical abstract

The invasive mysid shrimp *Limnomysis benedeni* exhibits a shift in food preference from zooplankton to microphytoplankton with increasing biomass of the latter. This suggests a remarkable feeding flexibility in *L. benedeni* with implications for the species' impact on community structure and ecosystem functioning.

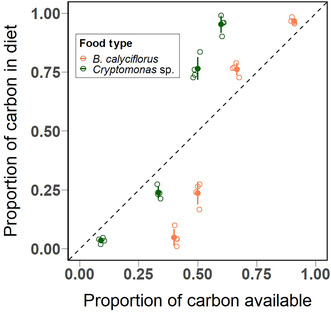

## 1 INTRODUCTION

Diet shift and food selectivity are among the most important aspects to understand the impact of a predator on community structure and food web functioning (Greene, 1983; Sailley et al., 2015). Food selection is influenced by multiple variables such as the predator's ontogenetic stage, food availability and size, and environmental factors such as temperature (Divoky et al., 2021; Graeb et al., 2006; Greene, 1986). According to the optimal foraging theory, the diet preference of an animal is influenced by maximising net energy gain and minimising the cost of obtaining the food (Gooding & Harley, 2015; Krebs et al., 1977). At the same time, foraging behaviour can be adjusted in response to changes in food density (Murdoch & Oaten, 1975). Usually, predators choose habitat patches with high food availability as it can increase the chances of successful predation events (Ioannou et al., 2009; Wellenreuther & Connell, 2002). Besides quantity, diet preference can also be influenced by the nutritional quality of their food (Mayntz et al., 2005; Schmidt et al., 2012). Along this trade‐off, consuming smaller and less nutritious food may only be beneficial if it is present in large quantities and/or if the higher quality food is difficult to capture (Langerhans et al., 2021). Omnivores can show higher foraging flexibility and may switch between different diet items depending on their availability. Their ability to exploit more than one trophic level (Pimm & Lawton, 1978) allows them to affect the dynamics of an ecosystem through multiple pathways. Understanding the feeding behaviours of omnivores, especially invasive species, can provide insight into their functional roles and be used to better predict population and ecosystem dynamics.

The spread of multiple Ponto‐Caspian invaders in numerous European brackish and freshwater habitats and more recently in North American Great Lakes received considerable attention in the past decades (Bij de Vaate et al., 2002; Reid & Orlova, 2002; Ricciardi & MacIsaac, 2000). The invasion was initially facilitated via unintentional introduction such as migration through artificial canals, reservoirs acting as stepping stones on rivers, and passive transport by ships (Jażdżewski, 1980). The expansion was further supported by intentional introduction to improve the fish food resources (Karpevich, 1975). Crustaceans, specifically, amphipods, cladocerans, and mysids are among the most successful Ponto‐Caspian invaders (Leppäkoski et al., 2002; Leppäkoski & Olenin, 2001; Ricciardi & Rasmussen, 1998). They can influence native communities by eliminating native species, decreasing functional diversity, and ultimately altering the energy flow in ecosystems (Dick & Platvoet, 2000; Jazdzewski et al., 2004; Ketelaars et al., 1999; Ojaveer et al., 2002).

Mysid shrimps, including species such as *Hemimysis anomala*, *Limnomysis benedeni*, *Katamysis warpachowskyi*, and *Paramysis lacustris*, have become prominent Ponto‐Caspian invaders. These species have successfully established in Europe (Audzijonyte et al., 2008, 2009; Borza et al., 2019; Wittmann, 2008), while *H. anomala* also invaded the North American Great Lakes (Audzijonyte et al., 2008). Traits such as wide environmental tolerance, omnivory, high annual number of generations, and related ability to get established quickly in new habitats likely contributed to their invasive success (Borza, 2014; Borza et al., 2017). Mysid shrimps are small, generally omnivorous crustaceans found in both marine and freshwater environments (Mauchline, 1980). By feeding on organisms from multiple trophic positions (e.g., phytoplankton and zooplankton), and representing food for higher trophic levels such as fish, they occupy key positions in their ecosystems (Arrhenius & Hansson, 1993; Rakauskas, 2019). Therefore, their establishment and changes in abundance can have cascading effects on other organisms in the ecosystem (Kiljunen et al., 2020). There are numerous studies exploring the feeding ecology of mysid shrimp species in their native areas (Fulton, 1982; Nordin et al., 2008; O'Malley & Bunnell, 2014). Less information is available on the feeding ecology of Ponto‐Caspian mysid shrimps in invaded ecosystems. More studies are clearly needed to be able to predict their impacts on the native communities. Quantifying the shift between herbivory and carnivory as a function of different food types and densities can be an important first step to achieve these goals. *Limnomysis benedeni* is among the most widespread mysid shrimps in European river and lake ecosystems (Audzijonyte et al., 2009; Borza, 2014; Borza et al., 2011; Wittmann, 2007). It feeds on small‐sized zooplankton and, smaller particles such as algal cells as well (Hanselmann et al., 2013). Earlier studies suggested that it may perform food density‐dependent diet switching between phytoplankton and zooplankton, with a potentially significant impact on plankton community structure and dynamics (Fink et al., 2012). However, the occurrence and direction of this diet shift were so far not quantified and studied in detail.

With this study, we aim to achieve a more quantitative understanding of the shift in diet preference of *L. benedeni* according to the changes in the availability of alternative food types. To do so, we compared the ingestion rates of *L. benedeni* on food mixtures containing a rotifer and a microalga, across increasing algal biomass. We expected *L. benedeni* to preferentially feed on the larger and highly nutritious food items (i.e., rotifers) as it was shown for other omnivorous crustaceans (Gulati & Demott, 1997; Meunier et al., 2016; Nejstgaard et al., 1997; Nejstgaard, Hygum, et al., 2001) and only switch to phytoplankton when present at sufficiently high biomass, and thus reducing predation on rotifers at high phytoplankton abundances.

## 2 MATERIALS AND METHODS

### 2.1 Study organisms and culturing conditions

Two different types of food, an alga and a microzooplankton species, were used in the experiment. *Cryptomonas* sp. (strain 26.80 of the SAG Culture Collection, originally isolated from Lake Windermere, UK) was grown in WC medium (Guillard, 1975) enriched with vitamin B12 (0.135 g L^−1^) (recipe available at UTEX culture collection of algae, Texas website). The *Cryptomonas* sp. cultures were grown in batch cultures and used during their exponential growth phase (ensured by regular refreshment of the medium) for experiments. The rotifer *Brachionus calyciflorus* (obtained from AQ4Aquaristics, Braunschweig, Germany) were cultivated in aerated and filtered (by a JBL Cristal Profi e702 external aquarium filter) tap water and were fed by *Cryptomonas* sp. Individuals of the mysid shrimp *Limnomysis benedeni* were collected by hand‐netting in the littoral zone of Lake Balaton, Hungary, on March 2022. They were subsequently reared in lab aquaria at 20°C, under a 16:8 light: dark photoperiod, and gentle aeration. The population was fed with *Cryptomonas* sp. at saturating concentrations (>1 mg C L^−1^) three times per week. Three‐fourths of the volume (~3–4 L) of the water in the aquaria was replaced with fresh filtered tap water twice a week.

### 2.2 Feeding experiment

Prior to setting up the experiment, we measured the axial dimensions of 20 randomly selected individuals of each food type. These measurements were needed to calculate their individual biomasses and to set up the experimental carbon biomass concentrations. The measurements were obtained using an inverted microscope (Zeiss Axio Vert.A1) after the individuals had been preserved with 1% Lugol's iodine solution. The biovolume of *Cryptomonas* sp. was approximated using the formula for a prolate spheroid (Hillebrand et al., 1999). Afterwards, the carbon content of *Cryptomonas* sp. was estimated using the formula $\mathrm{Cc}\left(\text{in}\frac{\mathrm{pg}}{\text{cell}}\right)=0.109*V^{0.991}$, where *V* is the biovolume in μm^3^ (Weisse et al., 2001). The biovolume of *B. calyciflorus* was estimated using the formula *V_r_* (in μm^3^) = 4*π* × *l* × *w* ^2^/3, where *l* is the length and *w* is the width (in μm) of *B. calyciflorus* (Bottrell et al., 1976). The dry weight of *B. calyciflorus* was calculated by assuming it to be 10% of the wet weight, which was calculated using the assumption that 1 mm^3^ equals 1 mg (Pace & Orcutt Jr., 1981). The dry weight was then converted to carbon content using a conversion factor of 0.48 (Work et al., 2005).

*Limnomysis benedeni* specimens were pre‐acclimatised to the experimental conditions for 48 h before the feeding experiment. We kept them in 200 mL glass jars with 160 mL of filtered tap water in two water baths at a constant temperature of 21°C, with gentle aeration and a 16:8 h light:dark photoperiod. Each jar contained three individuals of *L. benedeni*, fed by a mixture of *Cryptomonas* sp. and *B. calyciflorus* at saturating densities (both at 1 mg C L^−1^, i.e., a total of 2 mg C L^−1^) during the first 24 h, followed by exposing them to the experimental conditions (see below) in the next 24 h. Similar‐sized adult *L. benedeni* were used and randomly distributed across the treatments. We applied a third water bath with the same treatments containing individuals of *L. benedeni* used as backup, in order to replace any dead individuals in the experimental jars to keep experimental biomass constant.

**FIGURE 1:**
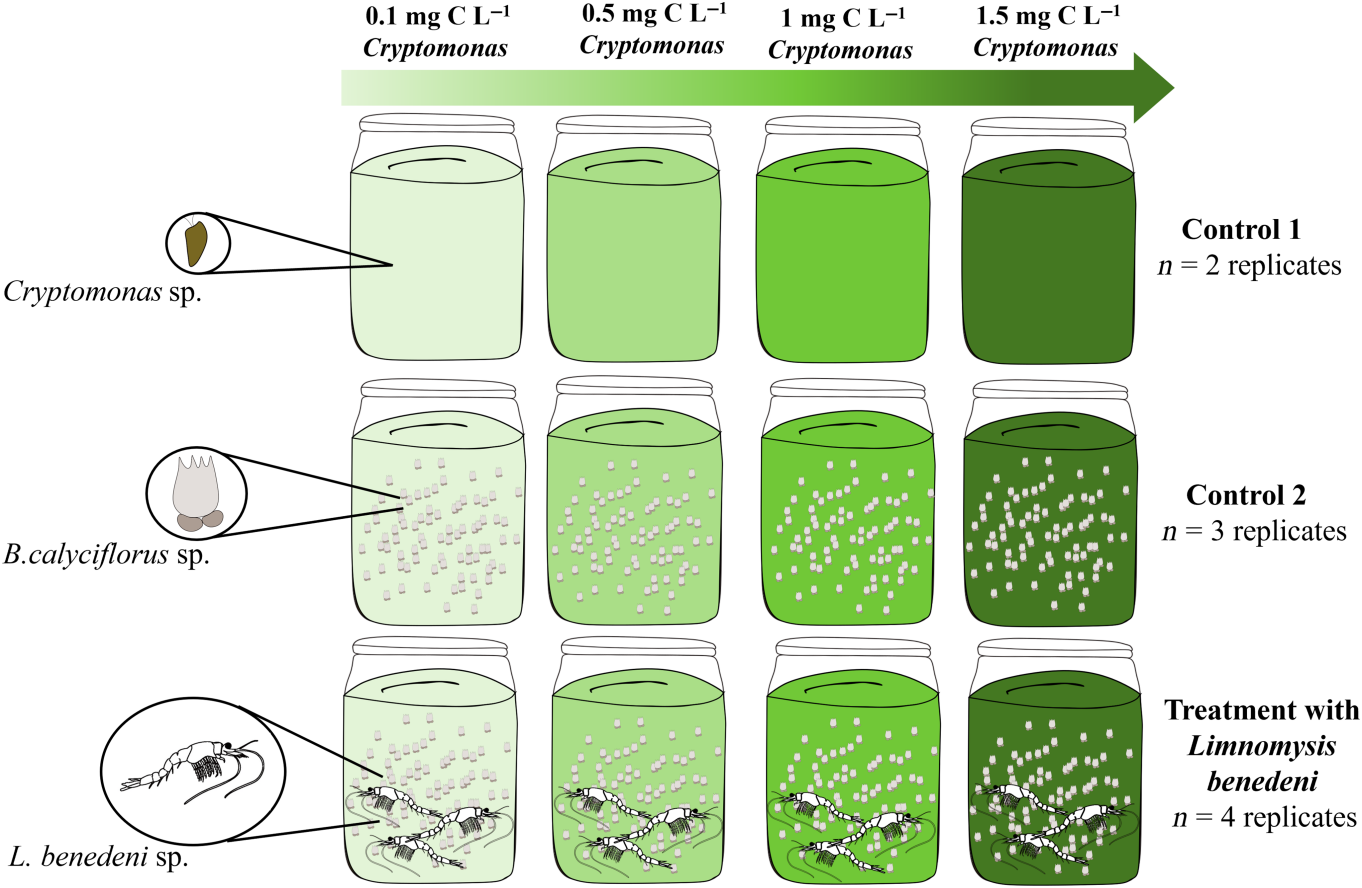
Schematic presentation of the experiment setup with the two types of controls (i.e., pure *Cryptomonas* sp. and *Cryptomonas* sp. with *Brachionus calyciflorus*) and the treatment with *Limnomysis benedeni*. Colour gradient represents the experimental treatment levels with increasing biomass of *Cryptomonas* sp. The biomass of *B. calyciflorus* was set to constant across all treatment levels, while the number of *L. benedeni* was set to 3 individuals per experimental jar.

After this 48‐h pre‐acclimatisation period, *L. benedeni* individuals were carefully rinsed in filtered tap water to remove any debris or food attached to them. To quantify grazing rates of *L. benedeni* on both food types, we run a feeding experiment consisting of four treatment levels with different *Cryptomonas* sp. biomasses (0.1, 0.5, 1, and 1.5 mg C L^−1^, corresponding to ~2000 k to 32,000 k cells mL^−1^), while *B. calyciflorus* biomass was kept constant (1 mg C L^−1^, ~3 individuals mL^−1^) across them (Figure 1). This setup was replicated four times. We applied two types of controls at each treatment levels: Control 1 containing pure *Cryptomonas* sp. to quantify its growth rate (applied in two replicates) and Control 2 containing both *Cryptomonas* sp. and *B. calyciflorus* to measure the consumption rate of *Cryptomonas* sp. by *B. calyciflorus* at different *Cryptomonas* sp. biomass treatments (applied in three replicates). The biomasses of food types in controls were the same as in the main treatments. The experiment was run for 14 h under constant dim light (4000 K LED, overall light intensity ~3 × 10^14^ photons cm^−2^ s^−1^). Other experimental conditions (i.e., medium, volume, temperature, aeration) were the same as described above for the pre‐acclimatisation period. The few dead individuals (three) of *L. benedeni* were replaced with individuals from the backup cultures within 15 min. At the end of the experiment, 1 and 10 mL samples were taken from each jar after thorough mixing to estimate the biomasses of *Cryptomonas* sp. and *B. calyciflorus*. The samples were fixed with 1% Lugol's iodine solution and counting was done using a stereomicroscope (Zeiss Stemi 305) using a Sedgwick rafter cell counter (S50, Graticules Optics, UK) for *Cryptomonas* sp. and a Petri dish with grids for *B. calyciflorus*.

For each food type (i.e., *Cryptomonas* sp. or *B. calyciflorus*), the specific growth rates (*k*_food_; h^−1^) were calculated using the formula suggested by Frost (1972):

$$k_{\text{food}}=\frac{\ln\left(C_{2}\right)-\ln\left(C_{1}\right)}{t_{2}-t_{1}}$$

The grazing coefficients (*g*; h^−1^) of *L. benedeni* for each food type (i.e., *Cryptomonas* sp. or *B. calyciflorus*) were calculated by:

$$g=k_{\text{food}}-\frac{\left[\ln\left(C_{2}^{*}\right)-\ln\left(C_{1}^{*}\right)\right]}{t_{2}-t_{1}}$$

where *C*_1_ and *C*_2_ are initial and final food abundances (individuals mL^−1^) in the control jars at times *t*_1_ and *t*_2_ and $C_{1}^{*}$ and $C_{2}^{*}$ are initial and final food abundances (individuals mL^−1^) in the jars with predators at times *t*_1_ and *t*_2_, respectively.

For each food type, filtration rates (*F*; mL individual^−1^ h^−1^) of *L. benedeni* were calculated using (Marin et al., 1986):

$$F=\frac{\mathrm{Vg}}{N}$$

where *V* is the volume of experimental jar (mL), *g* is the grazing coefficient (h^−1^) of *L. benedeni* and *N* is the number of *L. benedeni* in one experimental jar.

Ingestion rates (*I*; food item individual^−1^ h^−1^) of *L. benedeni* for each food type were calculated using the following formula (Marin et al., 1986):

$$I=\frac{F}{<C>}$$

where *F* is the filtration rate (mL individual^−1^ h^−1^) of *L. benedeni* for each food type and <*C*> is the mean concentration of each food type in the experimental jar.

The mean concentration of each food type (<*C*>; individuals mL^−1^) was calculated using the following formula (Marin et al., 1986):

$$<C>=\frac{C_{2}^{*}-C_{1}^{*}}{\ln\frac{C_{2}^{*}}{C_{1}^{*}}}$$

where all variables are defined earlier.

The corrected grazing rate (*g*′; h^−1^) of *L. benedeni* needed to account for the grazing of *B. calyciflorus* on *Cryptomonas* sp. was done using the following formula (Nejstgaard, Naustvoll, & Sazhin, 2001):

$$g^{\prime }=g+k^{\prime }$$

where *k*′ is the correction factor for the loss of *Cryptomonas* sp. by the grazing of *B. calyciflorus*.

The correction factor (*k*′) was calculated using the following formula (Nejstgaard, Naustvoll, & Sazhin, 2001):

$$k^{\prime }=g^{\prime }\left(\frac{\bar{c}-{\bar{c}}^{*}}{\bar{c}}\right)$$

where $\bar{c}$ and ${\bar{c}}^{*}$ are the mean concentrations of *B. calyciflorus* in Control 2 jars and jars with *L. benedeni*, respectively.

### 2.3 Data analysis

We ran multiple models, including linear models, generalised linear models, and generalised additive models to test how different *Cryptomonas* sp. densities affect the ingestion rates of *L. benedeni* on *Cryptomonas* sp. and *B. calyciflorus*. Model selection was then based on the Akaike Information Criterion (AIC), which selected generalised additive models (GAM) to be the relative best‐fit models (Burnham & Anderson, 2004). Ingestion rates on *Cryptomonas* sp. or *B. calyciflorus* were used as response variables, plotted against treatment levels (i.e., biomass of *Cryptomonas* sp.) respectively with *k* = 4. The models (including *k* values) were selected via an AIC‐based model selection while model diagnostics was carried out via the gam.check() function of ‘mgcv’ package. We used the ‘anova.gam’ of the ‘mgcv’ R package to test the significance of the smooth term, treatment levels (Wood et al., 2016). Model assumptions (normality, heterogeneity of variances across treatments) were visually assessed via diagnostic plots (histogram of model residuals, residuals vs. fitted values, normal Q–Q plots) and no deviations were found. All statistical tests were performed using R studio version 4.1.1 (R Core Team, 2021) with the nlme, mgcv, and ggplot2 packages (Pinheiro et al., 2022; Wickham, 2009; Wood et al., 2016). Besides, to quantify the preference of *L. benedeni* for different food items with respect to their relative biomass, we calculated Ivlev's index (*e*_*i*_) for each food type across treatments (Ivlev, 1961; Jacobs, 1974). Here, a positive score indicates a preference for a particular food item while a negative score may indicate inaccessibility or avoidance of the food item (Jacobs, 1974). We also calculated and plotted the relative biomasses (mean of three replicates with 95% confidence intervals) ingested from each food type against their relative availability. Here, if the relative ingested biomass of a given food type falls above the 1:1 line indicates preferential feeding (while below means avoidance) and a change in preference can be used to reveal food switching (Cuthbert et al., 2018; Hughes & Croy, 1993).

## 3 RESULTS

**FIGURE 2:**
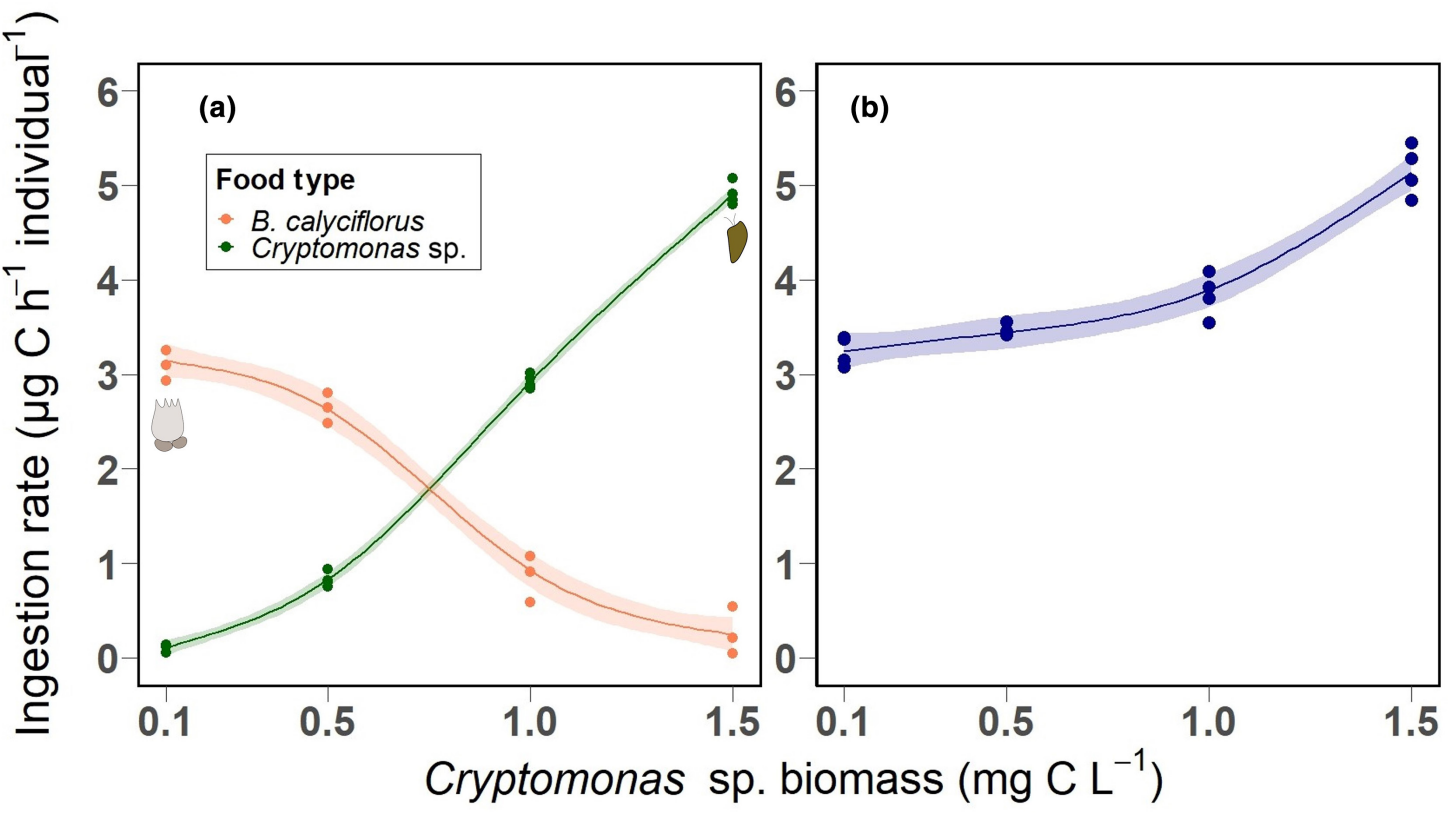
*Limnomysis benedeni* (a) ingestion rates (μg C h^−1^) on *Cryptomonas* sp. (dark green) and *Brachionus calyciflorus* (orange) across treatments (i.e., increasing initial biomass of *Cryptomonas* sp.). Solid lines represent fitted generalised additive models with coloured bands representing 95% confidence intervals. (b) Total ingested plankton biomass (sum of ingested *Cryptomonas* sp. and *B. calyciflorus* carbon biomasses, μg C L^−1^) with the increasing original biomass of *Cryptomonas* sp.

Ingestion rates of *L. benedeni* on *Cryptomonas* sp. increased in a slightly sigmoidal pattern with increasing *Cryptomonas* sp. biomass (Figure 2a). Parallel to this, ingestion rates on *B. calyciflorus* decreased following a clear sigmoidal pattern, with an abrupt decline occurring between *Cryptomonas* sp. concentrations 0.5 and 1.0 mg C L^−1^ (Figure 2a). The models explained 99.8% (adj. *R* ^2^ = .998) and 97.8% (adj. *R* ^2^ = .978) variance for *Cryptomonas* sp. and *B. calyciflorus* consumption as a function of *Cryptomonas* sp. biomass (Table 1). Consumption of *Cryptomonas* sp. or *B. calyciflorus* by *L. benedeni* were found to have a strong association with the experimental treatment levels (edf = 2.977 (for *Cryptomonas* sp.), *p* < .001, edf = 2.948 (for *B. calyciflorus*), *p* < .001). The total carbon biomass ingestion rate (i.e., the sum of ingested *Cryptomonas* sp. and *B. calyciflorus* carbon biomass) by *L. benedeni* increased with an increase in *Cryptomonas* sp. biomass. The relationship was non‐linear, with a steeper increase above 1 mg C L^−1^ than below (Figure 2b).

**TABLE 1:** Summary statistics of GAM models for the ingestion rate of *Limnomysis benedeni* on *Cryptomonas* sp. and *Brachionus calyciflorus* across treatment levels (different biomass of *Cryptomonas* sp.).

**Ingestion rate of *L. benedeni* on *Cryptomonas* sp.**					
Component	Term	Estimate	Std error	*t*‐Value	***p*‐Value**
Parametric components	(Intercept)	2.193	0.021	106.082	<.001

Component	Term	edf	Ref. df	*F*‐value	*p*‐Value
B Smooth terms	s(Density)	2.977	3.000	2750.681	<.001
Adjusted *R* ^2^: .998, Deviance explained 0.999					

**Ingestion rate of *L. benedeni* on *B. calyciflorus* sp.**					
Component	Term	Estimate	Std error	*t*‐Value	*p*‐Value
Parametric components	(Intercept)	1.736	0.046	37.633	<.001

Component	Term	edf	Ref. df	*F*‐value	*p*‐Value
B Smooth terms	s(Density)	2.948	2.998	221.599	<.001
Adjusted *R* ^2^: .978, Deviance explained 0.982.					

Abbreviations: edf, effective degree of freedom; Ref. df, reference degree of freedom.

The Ivlev's electivity index suggested a treatment‐specific preference for *Cryptomonas* sp. and *B. calyciflorus* (*e*_*i*_ > 0). At lower *Cryptomonas* sp. biomass (0.1 and 0.5 mg C L^−1^), index values showed a preference for *B. calyciflorus* (*e*_*i*_ = 0.02 and 0.08) versus *Cryptomonas* sp. (*e*_*i*_ = −0.09 to −0.6). However, at higher *Cryptomonas* sp. biomass (1 and 1.5 mg C L^−1^) the pattern shifted to the opposite, with higher *e*_*i*_ values for *Cryptomonas* sp. (0.18–0.25) than for *B. calyciflorus* (ranging from −0.2 to −0.9).

**FIGURE 3:**
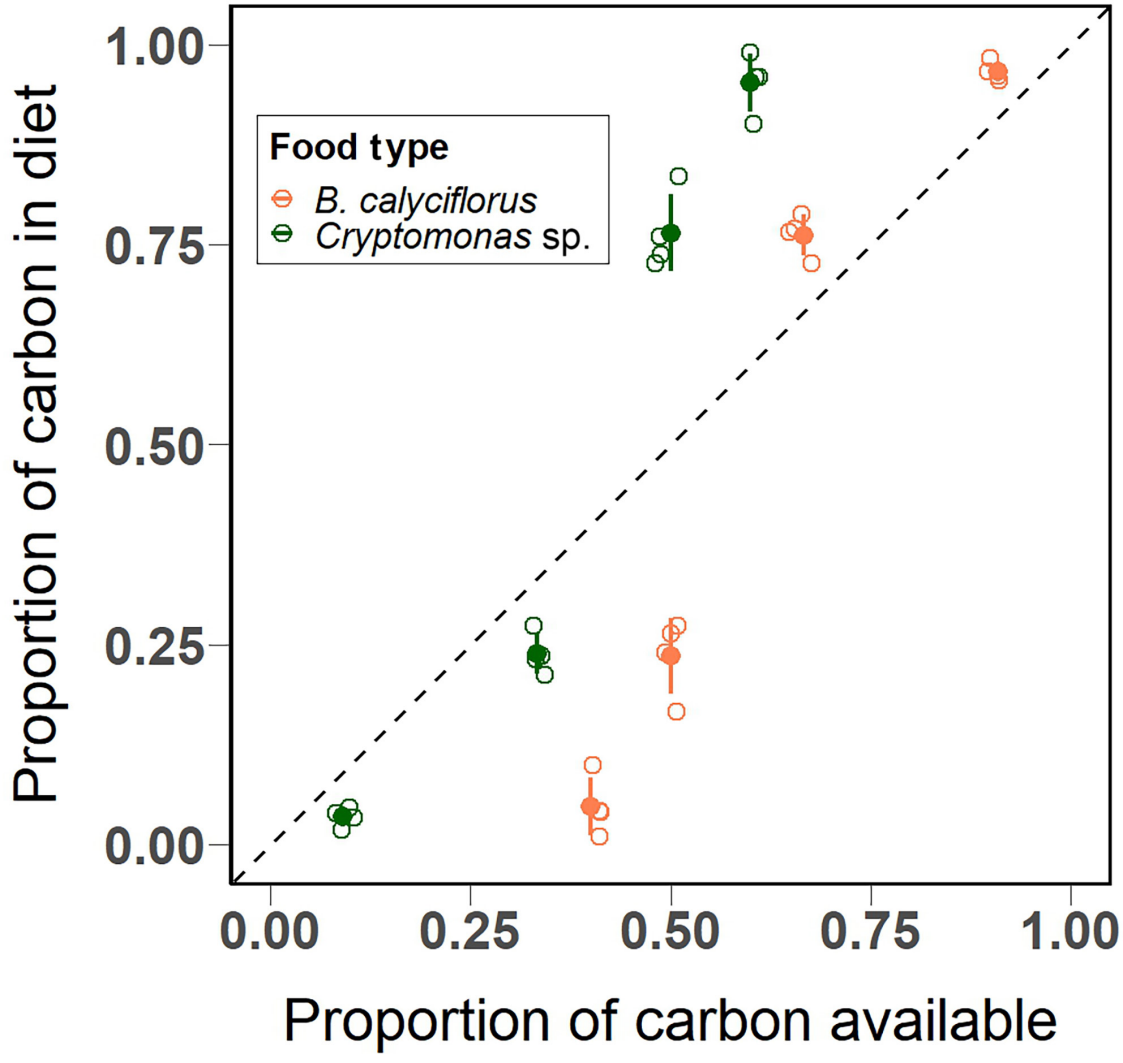
Relative contribution of food carbon biomass to the total ingested biomass by *Limnomysis benedeni* plotted as a function of the relative contribution of each food type to total available carbon biomass. Food types used are *Cryptomonas* sp. (dark green) and *Brachionus calyciflorus* (orange). Empty circles represent replicates, solid‐filled circles represent mean values while error bars indicate the 95% confidence intervals. The dashed 1:1 line represents where *L. benedeni* would be consuming food proportional to its availability, meaning random food uptake (neither preference nor avoidance).

The pattern of the relative contribution of *Cryptomonas* sp. and *B. calyciflorus* biomass to the total ingested biomass also confirmed treatment‐specific preferential feeding. We observed a shift in preference from *B. calyciflorus* to *Cryptomonas* sp. when the biomass of the latter was provided in equal or higher biomasses (i.e., treatment levels with 1 and 1.5 mg C L^−1^ *Cryptomonas* sp.) (Figure 3).

## 4 DISCUSSION

Our results clearly showed that the diet preference of *L. benedeni* depends on the relative (C) biomass concentration of food types, by shifting from *B. calyciflorus* to *Cryptomonas* sp. with increasing *Cryptomonas* sp. biomass. The patterns of consumption rates in response to relative food biomass differed between the food types suggesting slightly different functional responses for the rotifers and algae. While both resembled a sigmoidal pattern (Figure 2a), the ingestion rate on *B. calyciflorus* followed a type III functional response more clearly, which usually indicates either food switching or efforts to modify foraging behaviour in response to the food density (Holling, 1959; Kiørboe et al., 2018; Thomas et al., 1996). In the case of *Cryptomonas* sp. after a slower increase at low densities, the pattern was closer to linear and did not show saturation within the tested biomass range during the experiment, overall resembling a type I functional response (Holling, 1959), suggesting that consumption of *Cryptomonas* sp. is primarily a function of density.

The decreasing consumption rates on *B. calyciflorus* with increasing *Cryptomonas* sp. concentration illustrated how the presence of alternative food items in sufficient densities can lead to decreased predation pressure on another type of food. If more than one alternative food source is present, predators can select based on prey‐specific encounter rates to optimise foraging time and energy intake (Krebs et al., 1977). Specifically, the relative attack rate is a function of relative density and the switching can happen only if the likelihood of attacking the last eaten species is higher than attacking other species (Oaten & Murdoch, 1975). In our case, the cost‐to‐benefit ratio changes in favour of *Cryptomonas* sp. when it is provided at higher densities. Indeed, we found that *Cryptomonas* sp. consumption increased in comparison to *B. calyciflorus* consumption for biomass equal to or higher than 1 mg C L^−1^. This is also supported by Ivlev's index results which point towards the flexibility in the feeding behaviour of *L. benedeni* depending on *Cryptomonas* sp. density. The Ivlev's indices indicate a preference for *B. calyciflorus* when the latter was present in low biomasses, and vice versa with high *Cryptomonas* sp. biomass.

Predators typically select their diet based on size, nutritional quality, and escape responses of the prey (Cotonnec et al., 2001; Frost, 1972; Viitasalo & Rautio, 1998). They would try to increase their fitness by acquiring food with easier access and lower cost‐to‐benefit ratio (MacArthur & Pianka, 1966; Pyke et al., 1977; Stephens & Krebs, 1986). Earlier studies suggest that *L. benedeni* is not an optically‐oriented predator but selects its food based on size (Fink et al., 2012). Here we fed *L. benedeni* with two types of food with very different sizes. Purely from a size‐based perspective, feeding on the smaller food item is only beneficial when available in sufficient densities. This was confirmed here, with *L. benedeni* showing a gradual shift to the smaller‐sized food with its increasing quantity.

Food nutritional quality can also affect the development of consumers and influence their dietary choices (Gulati & Demott, 1997; Meunier et al., 2016). Algae generally have a higher carbon‐to‐nutrient ratio than animal food (Elser et al., 2000; Sterner & Hessen, 1994). In the case of the strain we used, *Cryptomonas* SAG 26.80, a C:P ratio of ~150 and a C:N ratio of ~22 were reported during the exponential growth phase (i.e., when nutrients are not limiting growth; Vad et al., 2020). In the present experiment, we also used and harvested it during exponential growth phase and therefore the elemental ratio can be expected to be similar. For *B. calyciflorus*, C:P ratios are relatively constant around ~92, while C:N are ~4 (Jensen & Verschoor, 2004), which is very close to the body stoichiometry of omnivorous crustaceans such as mysids (C:P ~ 90 and C:N ~ 4) (Arbačiauskas et al., 2013). Therefore, based on a purely elemental stoichiometric point of view (Laspoumaderes et al., 2010), *B. calyciflorus* would be the preferred food. The higher C to nutrient ratios of algae may explain the pattern we found in the case of total ingested carbon biomass, which instead of steadily increasing showed an accelerating increase at the highest *Cryptomonas* sp. biomass level. This may indicate that *L. benedeni* needs to increase overall food uptake when feeding on algae, to obtain sufficient amounts of nutrients.

Other factors such as essential fatty acids (Ahlgren et al., 1990; Brett & Müller‐Navarra, 1997; Ramlee et al., 2021; Trommer et al., 2019), or vitamins (Fridolfsson et al., 2018, 2019; Hessen, 1992) can also influence food quality. *Cryptomonas* sp. is considered to be a high‐quality algal food source based on the high cellular content of essential fatty acids (Von Elert & Stampfl, 2000; Weers & Gulati, 1997). Consequently, the results may differ if the autotrophic food quality is less cost‐efficient for the predator than the heterotrophic food. For instance, had a ‘less favourable’ algal food source, such as green algae, been used, the preference for *B. calyciflorus* might have been more pronounced. Nonetheless, a preference for green algae (*Chlamydomonas* sp.) over zooplankton has been observed previously at an algal biomass of 0.3 mg POC L^−1^ as well (Fink et al., 2012).

Given that our study is based on a short‐term experiment, there are some aspects in which its results might differ from patterns arising in natural communities. For instance, the container size in laboratory studies is a factor that may influence predator behaviour (Bergström & Englund, 2004; Toonen & Fu‐Shiang, 1993). Therefore, a small‐sized jar could have increased the competition among *L. benedeni* thus forcing them to choose *Cryptomonas* sp., a ‘costly food’, to consume maximum food to maintain their energy budget. The densities of *L. benedeni* in their natural habitat, e.g., Lake Balaton, can differ a lot depending on the season, wind conditions and microhabitat type, ranging from zero to several thousand individuals m^−2^ (Szalontai, 2008). We chose the density of 3 *L. benedeni* per experimental jar (1060 individuals m^−2^, hence representing a realistic density value) after a few trials. We ensured that the change in food concentration will be enough to obtain robust estimation of ingestion rates in 14 h and they did not show aggressive behaviour due to container size. In addition, in this feeding experiment, we did not include predators of *L. benedeni*, though in natural ecosystems optimal foraging strategy is based on a tradeoff between nutritional needs and simultaneously minimising the risk of predation as described in the unified foraging theories (Mangel & Clark, 1986). Experiments with another trophic level (e.g., small fish), carried out in larger mesocosms, could therefore provide more specific predictions on these tradeoffs in the future, including longer‐term effects on ecosystem stability.

Despite these limitations, our results provide important implications for plankton community dynamics in natural ecosystems. Depending on the relative biomasses of phyto‐ and zooplankton, *L. benedeni* may act as predators or competitors of zooplankton, being intraguild predators of the latter. By always suppressing the more abundant planktonic prey, they may reduce the amplitude of predator–prey oscillations, thereby contributing to ecosystem stability. However, there are certainly other possible scenarios, as illustrated by the effects of other invasive omnivorous Ponto‐Caspian mysids. For example, *Hemimysis anomala* and *Paramysis lacustris* were both found to contribute to the alteration of trophic pathways in their invaded habitats, due to their strong predatory impact on zooplankton and benthic macroinvertebrate communities (Evans et al., 2018; Ketelaars et al., 1999; Rakauskas, 2019). For a better understanding of the potential effects of omnivorous mysids on trophic cascades and food web stability, studying their feeding mechanisms and diet selection is of high importance. Field observations coupled with laboratory and mesocosms studies could contribute to understanding the mechanisms underlying community and ecosystem‐level effects of these widespread (and still spreading) invasive species.

## AUTHOR CONTRIBUTIONS

**Varsha Rani:** Conceptualization (equal); data curation (lead); formal analysis (equal); methodology (equal); writing – original draft (lead); writing – review and editing (equal). **Zsófia Horváth:** Conceptualization (supporting); formal analysis (equal); supervision (supporting); writing – review and editing (equal). **Jens C. Nejstgaard:** Conceptualization (equal); methodology (supporting); writing – review and editing (equal). **Ádám Fierpasz:** Methodology (supporting); writing – review and editing (supporting). **Károly Pálffy:** Formal analysis (equal); writing – review and editing (equal). **Csaba F. Vad:** Conceptualization (equal); data curation (supporting); formal analysis (equal); funding acquisition (lead); methodology (equal); resources (lead); supervision (lead); writing – original draft (lead); writing – review and editing (equal).

## CONFLICT OF INTEREST STATEMENT

The authors declare no conflict of interest.

## Data Availability

The data used to draw graphs in the study is available on Dryad https://datadryad.org/stash/share/xURDH3d1npo2cOZhJ0oOr3EIF5Lxc2p1u2d0Zf1YE0w.
