# Correction to “Food density drives diet shift of the invasive mysid shrimp, *Limnomysis benedeni*”

**DOI:** 10.1002/ece3.70033

**Published:** 2024-07-18

Rani, V., Horváth, Z., Nejstgaard, J. C., Fierpasz, Á., Pálffy, K., & Vad, C. F. (2024). Food density drives diet shift of the invasive mysid shrimp, *Limnomysis benedeni*. *Ecology and Evolution*, 14, e11202. https://doi.org/10.1002/ece3.11202

**Error description**:

The formula in Section 2; Material and Methods, Subsection 2.2; Feeding experiment, page 3, 4th equation in right‐hand side column (in PDF version) reads

“Ingestion rates (*I*; food item individual^−1^ h^−1^) of *L. benedeni* for each food type were calculated using the following formula (Marin et al., 1986):

$$I=\frac{F}{<C>},$$

where *F* is the filtration rate (mL individual^−1^ h^−1^) of *L. benedeni* for each food type and <*C*> is the mean concentration of each food type in the experimental jar.”

It should be replaced with

“Ingestion rates (*I*; food item individual^−1^ h^−1^) of *L. benedeni* for each food type were calculated using the following formula (Marin et al., 1986):

I=F<C>,

where *F* is the filtration rate (mL individual^−1^ h^−1^) of *L. benedeni* for each food type and <*C*> is the mean concentration of each food type in the experimental jar.”

This correction does not affect the results or outcome of the experiment in any way since the calculations were done using the correct formula.

We apologize for this error.

